# Risk factors for symptomatic rotator cuff tears: a retrospective case–control study

**DOI:** 10.3389/fmed.2023.1321939

**Published:** 2024-01-04

**Authors:** Jinlong Zhao, Lingfeng Zeng, Guihong Liang, Minghui Luo, Weiyi Yang, Jun Liu, Jianke Pan

**Affiliations:** ^1^State Key Laboratory of Traditional Chinese Medicine Syndrome/The Second Clinical College of Guangzhou University of Chinese Medicine, Guangzhou, China; ^2^The Second Affiliated Hospital of Guangzhou University of Chinese Medicine (Guangdong Province Hospital of Traditional Chinese Medicine), Guangzhou, China; ^3^The Research Team on Bone and Joint Degeneration and Injury of Guangdong Provincial Academy of Chinese Medical Sciences, Guangzhou, China; ^4^The Fifth Clinical Medical College of Guangzhou University of Chinese Medicine, Guangzhou, China; ^5^Guangdong Second Traditional Chinese Medicine Hospital (Guangdong Province Engineering Technology Research Institute of Traditional Chinese Medicine), Guangzhou, China

**Keywords:** symptomatic, rotator cuff tears, risk factors, logistic regression, retrospective

## Abstract

**Background:**

The incidence and diagnostic rate of rotator cuff tears (RCTs) have increased significantly. The purpose of this study was to investigate and analyze the risk factors for symptomatic RCTs to provide a basis for their prevention and treatment.

**Methods:**

We retrospectively analyzed the relevant clinical indicators of 193 patients with RCTs and 161 patients without RCTs hospitalized with shoulder pain as the main complaint from January 1, 2017, to August 31, 2021. Univariate analysis and multivariate *logistic* regression analysis were used to analyze the differences in potential risk factors between the two groups.

**Results:**

Univariate analysis revealed that age (*p* < 0.001), body mass index (BMI) (*p* = 0.036), hypertension (*p* < 0.001), coronary heart disease (*p* = 0.028), history of shoulder trauma (*p* < 0.001), hyperlipidemia (*p* = 0.025), type III acromion (*p* = 0.012) and critical shoulder angle (CSA) (*p* < 0.001) increased the risk of RCTs. Multivariate logistic regression analysis revealed that age ≥ 60 years (OR = 2.61, 95% CI = 1.23 to 5.12), CSA ≥ 35° (OR = 4.24, 95% CI = 1.60 to 11.22), hypertension (OR = 2.34, 95% CI = 1.33 to 4.11) and history of shoulder trauma (OR = 5.20, 95% CI = 2.87 to 9.45) were independent risk factors for symptomatic RCTs.

**Conclusion:**

The results of this study showed that age ≥ 60 years, CSA ≥35°, hypertension and history of shoulder trauma are independent risk factors for symptomatic RCTs and can provide directions for further development of prevention and treatment strategies. Future studies need to clarify the mechanism underlying the association between these risk factors and symptomatic RCTs.

## Background

With the accelerated aging process and the rapid development of diagnostic imaging techniques, the incidence and diagnostic rate of rotator cuff tears (RCTs) have increased significantly (1). The incidence rate of NAFLD in RCTs is 7–72% (1, 2), with an average incidence rate of 20.7% in the general population (1). RCTs account for 5 to 40% of all shoulder diseases, and 30 to 70% of shoulder pain problems are directly related to rotator cuff-related tears (3). RCTs can be divided into small tears, medium tears, large tears and very large tears according to the size of the tear (4). The treatment methods for RCTs mainly include drug treatment, physical therapy and surgical treatment, but the current treatment methods have objective adverse reactions or defects (5, 6). One of the more serious adverse reactions to rotator cuff surgical repair is postoperative retearing, and studies have shown that the retear rate can reach 31% 6 months after rotator cuff repair (7, 8), which is undoubtedly an enormous economic and psychological burden. Thus, this topic is highly valuable for interventions in people with RCTs to change the risk factors and reduce the burden caused by RCTs.

In recent years, research on the risk factors for RCTs has gradually increased; however, there are considerable differences in the conclusions of different studies, and there is also a lack of research on the occurrence of RCTs in the Chinese population. Therefore, we designed a retrospective case–control study and applied logistic regression analysis to explore the risk factors for symptomatic RCTs, which may be conducive to early evaluation of the population prone to RCTs and provide a theoretical basis for clinical prevention and treatment.

## Research design

### Research subjects and groups

**Figure 1:**
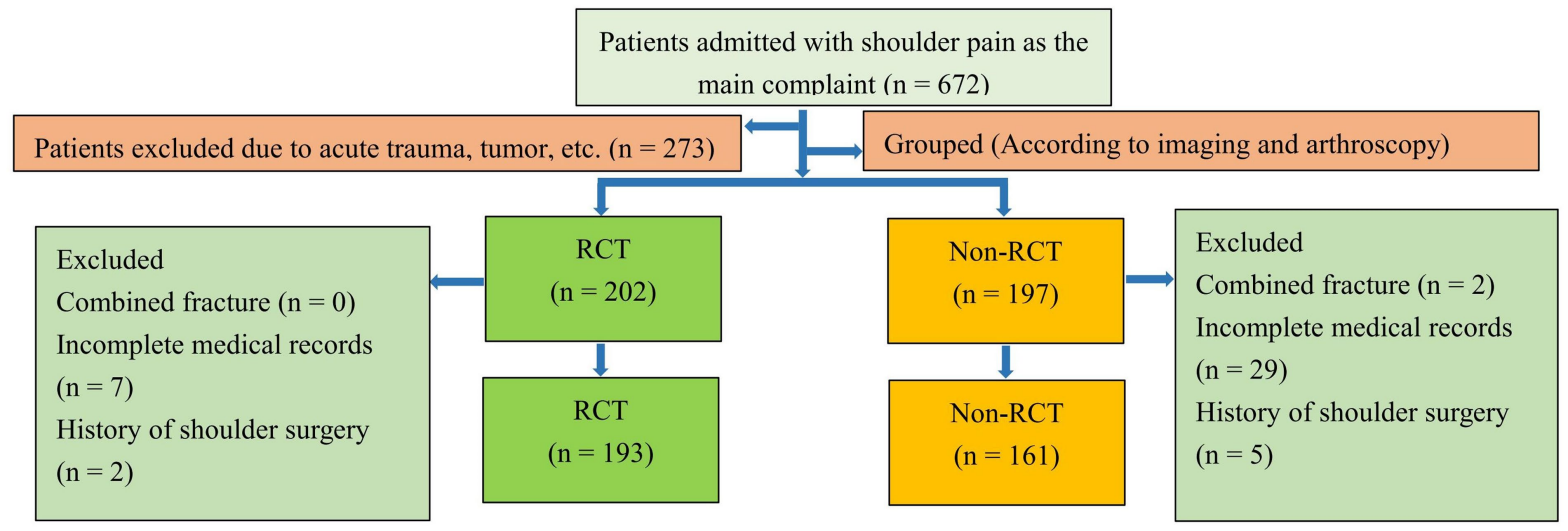
Flow chart of case screening and grouping.

We retrospectively analyzed patients hospitalized in the Department of Orthopedics at Guangdong Hospital of Traditional Chinese Medicine with “shoulder pain” as the main complaint from January 1, 2017, to August 31, 2021. Patients were divided into an RCT group and a non-RCT group according to the presence of a tear in the rotator cuff. This study was approved by the ethics committee of Guangdong Hospital of Traditional Chinese Medicine (number: YE2021-298-01). All the data used in our study were anonymous, and the requirement for informed consent was therefore waived (9). The process of patient screening and grouping is shown in Figure 1.

### Diagnostic criteria

In the RCT group, an RCT could be clearly diagnosed by MRI, MRA, ultrasound and other imaging examinations or arthroscopic exploration; in the non-RCT group, no RCT was found under imaging examination or arthroscopy.

### Inclusion criteria

1) Patients were admitted with “shoulder pain” as the main complaint, but the severity of shoulder pain or the stage of RCT did not have strict restrictions; 2) patients met the diagnostic criteria and had at least one objective diagnostic basis (imaging examination or arthroscopic exploration); 3) patients were aged ˃18 years; and 4) patients had complete medical records.

### Exclusion criteria

1) Patients with a previous history of shoulder surgery; 2) patients with acute shoulder trauma; 3) patients in whom an RCT was combined with other injuries, such as fracture; or 4) patients who were long-term bedridden or wheelchair-bound.

### Data collection

The data of patients who met the inclusion criteria were retrieved from the electronic medical records system of Guangdong Hospital of Traditional Chinese Medicine and collected. The potential risk factors included age, body mass index (BMI), smoking status, alcohol consumption, hypertension, type 2 diabetes mellitus, coronary heart disease, history of shoulder trauma, critical shoulder angle (CSA), type III acromion, white blood cell count (WBC), hemoglobin (HB), hyperlipidemia, and hyperuricemia. For the above data, the first measurements following admission were used.

### Statistical methods

SPSS 24.0 software (SPSS, Chicago, IL, United States) was used to analyze the data. The count data are expressed as frequencies and percentages. The contingency table data were analyzed with the *chi*-square test (or Fisher’s exact test). The normally distributed data are expressed as the mean ± SD, and an independent sample *t test* was used for intergroup comparisons. If the measurement data did not conform to a normal distribution or the variance was uneven, they were expressed as the median and interquartile range [*M* (*P*25, *P*75)], and the nonparametric *Wilcoxo*n rank sum test was used for comparisons between groups. The nonparametric *Mann–Whitney U* test was used for grade data. All variables with statistical significance in univariate analysis were analyzed by unconditional stepwise logistic regression. The alpha level was *α* = 0.05.

## Results

### Basic information

A total of 354 patients were included in this study, including 193 patients in the RCT group and 161 patients in the non-RCT group. Among the 354 patients, the minimum age was 33 years, and the maximum age was 85 years. There were 71 males and 122 females in the RCT group and 56 males and 105 females in the non-RCT group.

### Univariate analysis of risk factors for RCTs

Univariate analysis revealed no significant differences in sex, smoking status, alcohol consumption, type 2 diabetes status, hyperuricemia status, or WBC or Hb levels between the RCT group and the non-RCT group (*p* > 0.05). There were significant differences in age (*p* < 0.001), BMI (*p* = 0.036), hypertension (*p* < 0.001), coronary heart disease (*p* = 0.028), history of shoulder trauma (*p* < 0.001), hyperlipidemia (*p* = 0.025), or type III acromion (*p* = 0.012) and CSA (*p* < 0.001). The univariate analysis results are shown in Table 1.

**Table 1:** Univariate analysis of risk factors for rotator cuff tears.

Characteristic	Non-RCT (*n* = 161)	RCT (*n* = 193)	Univariate analysis	
			Χ^2^/t/Z-test	*p*
Sex (*n*, %)			0.153	0.70
Male	56 (34.78)	71 (36.79)		
Female	105 (65.22)	122 (63.21)		
Age (years, mean ± sd)	56.92 ± 9.01	61.95 ± 9.60	−5.05	0.000
Age (years, *n*, %)			−4.71	0.000
<50	35 (21.74)	21 (10.88)		
50 ~ 59	63 (39.13)	48 (24.87)		
≥60	63 (39.13)	124 (64.24)		
BMI [kg/m^2^, M (P75, P25)]	23.40 (25.39, 21.28)	24.02 (26.20, 22.06)	−2.26	0.024
BMI (kg/m^2^, *n*, %)			−2.10	0.036
<18.5	9 (5.59)	6 (3.11)		
18.5 ~ 23.99	86 (53.42)	89 (46.11)		
24 ~ 27.99	54 (33.54)	75 (38.86)		
≥28	12 (7.45)	23 (11.92)		
Smoking (*n*, %)			0.001	0.97
Yes	19 (11.80)	23 (11.92)		
No	142 (88.20)	170 (88.08)		
Alcohol drinking (*n*, %)			1.541	0.22
Yes	10 (6.21)	19 (9.84)		
No	151 (93.79)	174 (90.16)		
Hypertension (*n*, %)			14.19	0.000
Yes	30 (18.63)	71 (36.79)		
No	131 (81.37)	122 (63.21)		
Type 2 diabetes mellitus (*n*, %)			2.56	0.11
Yes	20 (12.42)	36 (18.65)		
No	141 (87.58)	157 (81.35)		
Coronary heart disease (*n*, %)			4.83	0.028
Yes	3 (1.86)	13 (6.74)		
No	158 (98.14)	180 (93.26)		
History of shoulder trauma (n, %)			40.18	0.000
Yes	18 (11.18)	80 (41.45)		
No	143 (88.82)	113 (58.55)		
Hyperuricemia (*n*, %)			3.06	0.08
Yes	13 (8.07)	27 (13.99)		
No	148 (91.93)	166 (86.01)		
Hyperlipidemia (*n*, %)			5.04	0.025
Yes	17 (10.56)	37 (19.17)		
No	144 (89.44)	156 (80.83)		
Type III acromion (*n*, %)			6.27	0.012
Yes	50 (31.06)	85 (44.04)		
No	111 (68.94)	108 (55.96)		
CSA [°, M (P75, P25)]	35.41 (39.10, 31.37)	38.00 (40.80, 34.67)	−4.78	0.000
CSA (*n*, %)			−4.45	0.000
<30°	22 (13.66)	7 (3.63)		
30–35°	55 (34.16)	44 (22.80)		
≥35°	84 (52.17)	142 (73.58)		
WBC [10^9^/L M (P75, P25)]	5.91 (6.88, 5.05)	6.11 (7.40, 5.30)	−1.52	0.128
HB[g/L, M (P75, P25)]	136.00 (145.00, 128.00)	135.00 (145.50, 126.00)	−0.628	0.53

### Multivariate logistic regression analysis of risk factors for RCTs

**Figure 2:**
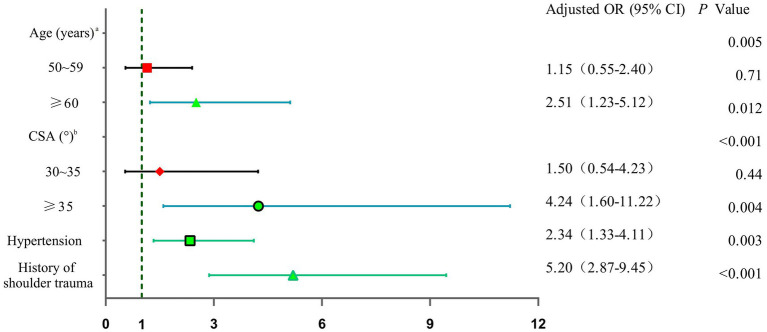
Forest plot showing the independent predictors of rotator cuff tears. a: age < 50 as reference; b: CSA <30° as reference.

Variables with a *p* value <0.05 in univariate analysis were considered to constitute the independent variable (X), and RCTs were considered to constitute the dependent variable (Y). The above variables were included in the logistic regression model for analysis, and the odds ratio (OR) and 95% confidence interval (CI) were calculated. The results showed that age ≥ 60 (age < 50 as a reference) (odds ratio (OR) = 2.61, 95% CI = 1.23 to 5.12; *p* = 0.012), CSA ≥35° (CSA <30° as a reference) (OR = 4.24, 95% CI = 1.60 to 11.22; *p* = 0.012), hypertension (OR = 2.34, 95% CI = 1.33 to 4.11) and history of shoulder trauma (OR = 5.20, 95% CI = 2.87 to 9.45) were found to be independent risk factors for symptomatic RCTs. The logistic regression analysis results are shown in Table 2 and Figure 2.

**Table 2:** Multivariate logistic regression analysis of rotator cuff tears.

Factors	*β*	Standard Error	Wald	OR (95% CI)	*p*
Age (years)	–	–	10.73	–	0.005
< 50 (Reference)	–	–	–	1.00	–
50 ~ 59	0.14	0.38	0.14	1.15 (0.55–2.40)	0.71
≥ 60	0.92	0.36	6.38	2.51 (1.23–5.12)	0.012
CSA (°)	–	–	18.99	–	0.000
< 30 (Reference)	–	–	–	1.00	–
30 ~ 35	0.41	0.53	0.60	1.50 (0.54–4.23)	0.44
≥ 35	1.44	0.50	8.47	4.24 (1.60–11.22)	0.004
Hypertension	0.85	0.29	8.80	2.34 (1.33–4.11)	0.003
History of shoulder trauma	1.65	0.30	29.37	5.20 (2.87–9.45)	0.000
Constant	−2.01	0.54	13.72	0.13 (−)	0.000

## Discussion

Prior to our study, there was still a lack of data on the risk factors for RCTs in the Chinese population, and our study filled this gap. RCT is a musculoskeletal disease that seriously affects the quality of life of patients. An increasing number of studies have focused on the factors that may cause RCTs and have tried to clarify the risk factors for RCTs (10–12). At present, conclusions on these risk factors remain controversial (13), and relevant research in Chinese populations is lacking. The risk factors for symptomatic RCTs were assessed with univariate analysis and multivariate logistic regression analysis. Univariate analysis revealed that older age; higher BMI; hypertension; coronary heart disease; history of shoulder trauma; hyperlipidemia; type III acromion; and greater CSA could increase the risk of symptomatic RCTs. In this study, multivariate logistic regression showed that age ≥ 60 years, CSA ≥35°, hypertension and shoulder trauma were independent risk factors for symptomatic RCTs. Therefore, these factors can serve as predictive factors for the occurrence of shoulder pain in RCTs. In addition, health management of patients with modifiable factors, such as hypertension, may further improve the incidence of RCTs.

A higher BMI increases the risk of symptomatic RCTs. A case–control study involving 2,738 patients showed that patients in RCTs had a higher BMI, with an OR of 1.45 (95% CI = 1.24–1.69) (14). This study suggested that a higher BMI may be a manifestation of obesity and other metabolic syndrome diseases (such as diabetes, hypertension and hyperlipidemia) and suggested that these diseases affect the microvascular supply to the rotator cuff tendon, especially near the supraspinatus muscle, resulting in susceptibility to tears in the rotator cuff (14). Obesity may increase the release of proinflammatory cytokines and promote the oxidative stress response, resulting in damage to the rotator cuff (15, 16). The results of this study suggest that CHD can increase the risk in RCTs. Although few studies have focused on this topic at present, our findings also provide a direction for us to fully understand the risk factors for RCTs. The possibility of vascular damage to the rotator cuff is increased due to rotator cuff-related vascular damage (17, 18). Previous studies have confirmed that patients in RCTs have higher blood lipid levels, which is consistent with our results, but the mechanism of the effect of hyperlipidemia on RCTs still needs to be further studied (19, 20). An imaging study on the relationship between acromion shape and RCTs showed that the size of RCTs involving patients with type III acromions was significantly greater than that of patients with type I or II acromions, which indicates that the shape of the acromion affects not only the occurrence of RCTs but also the size of RCTs (21, 22).

Age ≥ 60 years, CSA ≥35°, hypertension and history of shoulder trauma were found to be independent risk factors for symptomatic RCTs. RCT is more often regarded as a degenerative muscle disease, so an increase in age is considered closely related to its occurrence (23). A study concluded that the risk of RCT was greater at older ages, with an OR of 2.44 (95% CI = 2.12–2.89) (11). Aging can lead to a decrease in muscle strength and the aging of microvessels around the rotator cuff, resulting in a decrease in rotator cuff strength and toughness (24). The results of this study showed that the risk of RCT in people older than 60 years was 2.6 times greater than that in people younger than 50 years, which provides a clear basis for identifying the risk factors for RCT in middle-aged and older adult people according to age. The CSA is a shoulder imaging index that has been studied more frequently in recent years. This difference is thought to be related to the findings of RCTs, but there are still many disputes (25, 26). A meta-analysis suggested that although the CSA can be reliably measured, the difference in the CSA between case and control groups ranges from very large to moderate or almost no difference, and given the objective heterogeneity of existing studies, it is difficult to obtain a deep understanding of the strength of the correlation between the CSA and RCTs (27). It is also thought that there is a significant correlation between full sleeve tears and the CSA (28). The reason supporting this view is that a larger CSA will put a greater mechanical load on the rotator cuff, which may directly lead to RCT (29). Gerber et al. (30) confirmed that when the same shoulder joint abduction and lifting activities are completed, especially in the initial stage of shoulder joint abduction, when the CSA is large, the overall strength of the deltoid muscle tends to lean toward shear force, and the supraspinatus muscle needs to bear excessive vertical force to compensate for increasing the pressure on the humeral head to balance the vertical force couple of the shoulder joint and maintain the stability of the shoulder joint rotation center. Therefore, if the supraspinatus muscle is in a high load state for a long time, it is prone to early degeneration and even full layer tearing (31). This study revealed that, compared with those with a CSA <30°, people with a CSA ≥35° have a greater risk of RCTs. These research data can provide scientific evidence for the clinical application of the CSA and confirm that a larger CSA is a risk factor for RCT. At present, the published literature is controversial regarding the relationship between hypertension and the risk of RCTs (13, 32). There are few studies on the mechanism of the internal relationship between hypertension and RCTs, so additional clinical and basic research is needed. The design of a cohort study to explore the incidence rate of RCTs in hypertensive and nonhypertensive populations and whether it is beneficial to reduce the incidence rate of RCTs by controlling hypertension are worthy of confirmation. In addition, exploring the effects of hypertension on the microanatomical structure and biomechanics of the rotator cuff will help us further understand the potential association between RCTs and hypertension. According to the results of this study, it is clear that the prevention or control of hypertension may reduce the risk of rotator cuff injury. A history of shoulder trauma is also considered a risk factor for RCT and has been widely recognized by clinicians (33); this finding is also supported by the reliable data in this study. A previous study suggested that work, including manufacturing, technical work, construction work and manual labor, may increase the risk of trauma, which in turn increases the risk of rotator cuff injury (34). RCT is common in the distal vascular supply of the supraspinatus tendon or infraspinatus tendon and is known as the “risk area” for rotator cuff injury. Therefore, the affected shoulder has been injured, resulting in chronic loss of blood supply and rotator cuff tears (33, 35).

### Limitations

Despite the findings of this study, there are inevitably the following limitations, which need to be further improved in the design of future research. This was a retrospective case–control study, which inevitably has the bias of clinical methodology. For example, there may be different imaging angles in shoulder X-ray positive films, resulting in differences in imaging measurement data. Therefore, all readers who interpret the imaging data in this study need to consider the objective clinical heterogeneity, which means that there may be measurement bias and reduce the credibility of the imaging data in this study. On the other hand, the patients included in this study were from a single-center sample in southern China, which may lack representativeness of the Chinese population. Future research on the risk factors for RCTs should include more rigorous, randomized, multicenter and large-sample clinical or cohort studies.

## Conclusion

The results of this study showed that age ≥ 60 years, CSA ≥35°, hypertension and history of shoulder trauma were independent risk factors for symptomatic RCTs. Future research needs to clarify the mechanism underlying the association between these risk factors and symptomatic symptoms through RCTs, which can provide direction for the further development of prevention and treatment strategies. Due to the limitations of the study design, it is necessary to carry out a large-sample, multicenter cohort study or clinical randomized controlled study in the future to verify the conclusions of the current study and explore other potential risk factors not involved in this study.

## Data availability statement

The original contributions presented in the study are included in the article/supplementary material, further inquiries can be directed to the corresponding authors.

## Ethics statement

The studies involving humans were approved by the ethics committee of Guangdong Hospital of Traditional Chinese Medicine (Number: YE2021-298-01). The studies were conducted in accordance with the local legislation and institutional requirements. Written informed consent for participation was not required from the participants or the participants’ legal guardians/next of kin in accordance with the national legislation and institutional requirements.

## Author contributions

JZ: Conceptualization, Data curation, Investigation, Methodology, Visualization, Writing – original draft, Writing – review & editing. LZ: Conceptualization, Data curation, Investigation, Methodology, Resources, Validation, Writing – original draft, Writing – review & editing. GL: Conceptualization, Data curation, Formal analysis, Investigation, Visualization, Writing – original draft, Writing – review & editing. ML: Data curation, Investigation, Validation, Writing – original draft, Writing – review & editing. WY: Conceptualization, Funding acquisition, Project administration, Supervision, Writing – original draft, Writing – review & editing. JL: Conceptualization, Funding acquisition, Project administration, Supervision, Writing – original draft, Writing – review & editing. JP: Data curation, Formal analysis, Investigation, Methodology, Visualization, Writing – original draft, Writing – review & editing.
